# *In Vitro* and *In Vivo* Evaluation of the Anticancer and Anti-inflammatory Activities of 2-Himachelen-7-ol isolated from *Cedrus Libani*

**DOI:** 10.1038/s41598-019-49374-9

**Published:** 2019-09-06

**Authors:** Andree Elias, Wassim N. Shebaby, Bilal Nehme, Wissam Faour, Bassem S. Bassil, Joelle El Hakim, Rita Iskandar, Nahia Dib-Jalbout, Mohamad Mroueh, Costantine Daher, Robin I. Taleb

**Affiliations:** 1 Department of Natural Sciences, Lebanese American University, Byblos, 1102 2801 Lebanon; 2 School of Medicine, Lebanese American University, Byblos, 1102 2801 Lebanon; 3 Faculty of Arts and Sciences, University of Balamand, PO Box 100, Tripoli, Lebanon; 4 School of Pharmacy, Lebanese American University, Byblos, 1102 2801 Lebanon

**Keywords:** Chemotherapy, Toxicology

## Abstract

*Cedrus libani* is a majestic evergreen tree native to the Mediterranean mountains of Lebanon, Syria and Turkey. In this study, the tree heart wood was extracted using hexane to produce *C. libani* oil extract (CLOE) as a dark oil. GCMS analysis of CLOE identified up to 30 compounds whereby 2-himachalen-7-ol (7-HC) was the most abundant (40%). 7-HC was isolated using column chromatography and the identity of the white crystalline solid was confirmed via NMR spectroscopy and X-Ray Crystallography. 7-HC demonstrated potent cytotoxic activity against several human cancer cell lines including brain (SF-268, IC_50_ 8.1 μg/mL) and colon (HT-29, IC_50_ 10.1 μg/mL; Caco-2, IC_50_ 9.9 μg/mL) with ovarian (Sk-OV-3, IC_50_ > 50 μg/mL) cells being the most resistant. However, while HT-29 displayed resistance to Cisplatin, 7-HC was 8–10 folds more potent. Co-treatment with 7-HC and Cisplatin showed a significant synergistic anti-proliferative effect against SF-268, HT-29 and Caco-2 cells. 7-HC also exhibited significant anti-inflammatory effect in formalin-induced paw edema in rats. Western blot analysis revealed that 7-HC displayed dose dependent inhibition of LPS-induced COX-2 protein expression in isolated rat monocytes. The present study demonstrates that 7-HC possesses promising anticancer and anti-inflammatory activities, and may serve as a lead molecule in cancer therapy.

## Introduction

Phytomedicine have been heavily utilized in the treatment and the chemoprevention of various cancer types, due to the pro-apoptotic and anti-proliferative activities of numerous plant-derived compounds^1^. Among the current anticancer medications, more than 60% of drugs approved for cancer treatment between 1940 and 2002 have either been natural products or formulations based on natural products; a rate higher than any other area of drug development^2^.

The Lebanese Cedar (*Cedrus libani, C. libani*) is a conifer species native to the Mediterranean mountains of Lebanon and Syria as well as the Toros Mountains of Turkey^3^. Ethnobotanical and traditional uses of the *Cedrus* genus traces back to ancient times whereby its essential oil was used in traditional medicine^4^. Phytochemical analysis of oils extracted from several *Cedrus* species revealed that steroids, procyanidins and terpenoids are the major constituents^5,6^. These phytochemicals have been shown to possess a wide range of biological activities, such as anticancer^7^, anti-inflammatory^8^ and antimicrobial effects^9^. The wood oil of *C. libani* was shown to possess potent antitumor effect against K562 human chronic myelogenous leukemia cells, as well as against multi-drug resistant leukemia cells^10,11^. Additionally, the *C. libani* wood oil induced erythroid differentiation at the terminal phase which is known to stimulate the expression of fetal globin genes^10^. Remarkably, the oil did not display cross-resistance, and as such this phenomenon elevates the stature of *C. libani* oils as a promising source of natural active compounds that may be formulated into agents used for the treatment of drug-resistant leukemia and refractory tumors^10^.

Inflammation is a host defense mechanism to eradicate invading pathogens and to initiate the healing process, but the excessive production of inflammatory mediators may cause injury to host cells along with chronic inflammation leading to neoplastic transformation and progression of malignancies^12^. During an inflammatory response, the pro-inflammatory biomarkers are excessively produced such as the reactive oxygen species (ROS), reactive nitrogen species (RNS), tumor necrosis factor-α (TNF-α) and PGE_2_^13^. In this context, the RNS such as nitric oxide (NO) is formed from inducible nitric oxide synthase (iNOS)^14^ and PGE_2_ is produced from arachidonic acid metabolites by COX-2^15^. PGE_2_ has been shown to enhance abnormal cell division, decrease apoptosis, increase angiogenesis, and promote tumor invasiveness and metastasis^16^. Other studies indicated that the overexpression of COX-2 is highly associated with the development of numerous types of epithelial cell-derived cancers including breast, skin, colon, lung, and prostate^17^. Furthermore, inhibition of inducible COX-2^18^ by non-steroidal anti-inflammatory drugs caused a decrease in angiogenesis, consequently their use can lead to inhibition of tumor progression and metastasis^19^. Generally, steroids and non-steroidal anti-inflammatory drugs are considered effective medications for prevention of ischemic events and treatment of pain, fever and inflammation. However, these drugs are associated with a significant risk of developing gastrointestinal or cardiovascular complications^20^. Therefore, the selective targeting of COX-2 activity or expression is of considerable clinical importance.

Preliminary studies in our lab demonstrated that *C. libani* wood oil exhibited potent anticancer activity against several human cancer cell lines. Analysis of the crude oil revealed that 2-himachalene-7-ol (himachalol; 7-HC) is a major constituent amounting to around 40% of the extract. The present study aims to isolate 7-HC and investigate its anticancer activity on various cancer cell lines as well as assess its *in vivo* and *in vitro* anti-inflammatory properties.

## Results

### Extraction and characterization of 2-himachelen-7-ol

**Figure 1:**
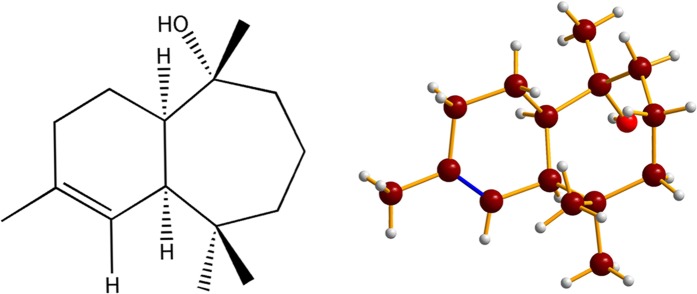
Structure and X-ray crystal structure of 7-HC.

*C. libani* wood was extracted using warm hexane to yield *C. libani* Oil Extract, CLOE) as a dark oil (1.95% yield). GCMS analysis of CLOE (Table 1) identified 30 compounds constituting around 83% of the oil extract with the remaining 17% constituting several unidentified compounds whereby none were found to be present in excess of 2%. Notably however, 7-HC (Fig. 1) was shown to be present at a staggering concentration of 40% of CLOE. In an attempt to isolate 7-HC, the oil was then subjected to silica-gel based column chromatography whereby three fractions (F1, F2 and F3) were obtained using a gradient mobile phase of hexane:ethyl acetate. 7-HC was eluted in F2 using 8:2 hexane:ethyl acetate as shown via TLC in which plates were stained with anisaldehyde and the sesquiterpene appeared as a pink spot with an *Rf* value of 0.78. The sesquiterpene was then recrystallized by adding acetonitrile to the F2 fraction to yield 7-HC as a white crystalline solid (97% purity; Fig. 1). The identity and structure of the major compound was confirmed as 2-himachelene-7-ol via GCMS analysis using the NIST11 and Wiley9 mass-spectral database and via ^13^C NMR spectroscopy (Supplementary Figs S1–S7).

**Table 1:** Gas Chromatography analysis of CLOE.

Peak	RT	% Area	Compound
1	4.597	0.0692	β**-**Pinene
2	14.73	2.9642	α-Himachalene
3	15.83	3.1818	γ-Himachalene
4	16.74	3.3695	β-Himachalene
5	17.2	0.3641	Butylated Hydroxytoluene
6	17.23	0.4236	Butylated Hydroxytoluene
7	18.26	0.3495	1,1,2,2,3,3-Hexamethylindane
8	20.82	0.8447	4,4-Dimethyl-3-(3-methylbut-3-enylidene)-2-methylenebicyclo[4.1.0]heptane
9	22.18	0.841	δ-Himachalene
10	23.24	40.2246	2-Himachalen-7-ol
11	23.76	2.7206	1H-Benzocycloheptene
12	25.53	7.4441	Dodecyl acrylate
13	34.25	0.9166	(S)-Phenol, 2-methyl-5-(1,2,2-trimethylcyclopentyl)
14	38.16	0.9471	Dibutyl phthalate
15	53.48	0.2794	Methyl isopimarate
16	53.66	0.5158	Abietadien-18-al
17	54.69	0.4301	Methyl levopimarate
18	55.48	0.125	Methyl abietate
19	55.91	0.3668	Tetracosane
20	57.02	0.5515	methyl 2,7,13 - abietatrienoate
21	57.08	0.3106	Octadecane
22	57.8	0.3232	Tricontamethylcyclopentadecasiloxane
23	57.97	0.1247	Eicosane
24	58.76	0.2048	Eicosane
25	59.48	2.7469	(Z)-9-Octadecenamide
26	60.1	0.2388	Tetracosamethyl-cyclododecasiloxane
27	65.36	2.3188	3β-Ergost-5-en-3-ol
28	67.21	7.8082	gamma Sitosterol
29	68.15	0.4482	(24 R)-Ergost-4-en-3-one
30	70.49	1.37	Stigmast-4-en-3-one

### Dose-dependent ***in vitro*** cytotoxicity of 7-HC

**Figure 2:**
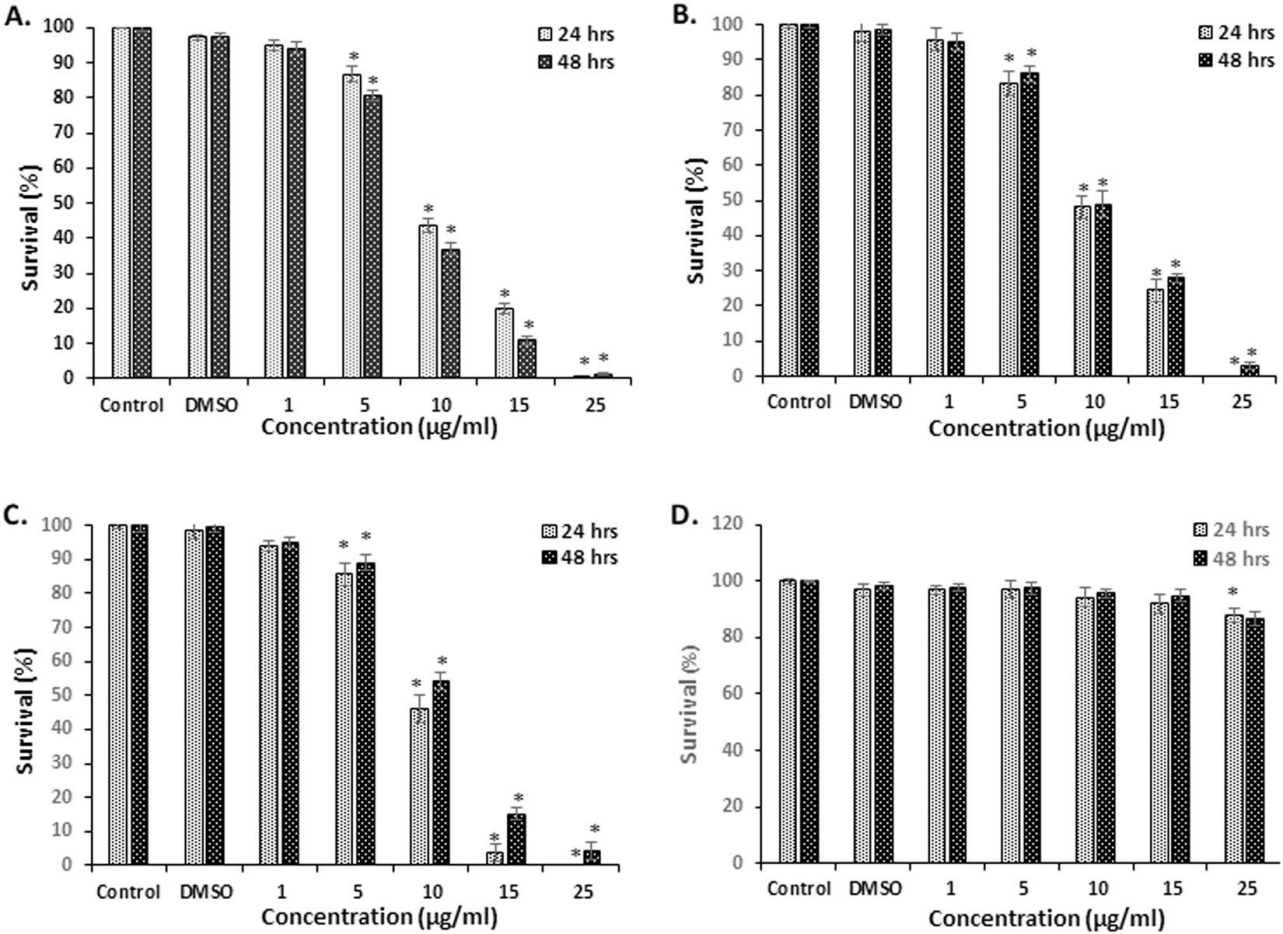
Cytotoxic effect of 7-HC on cell survival. SF-268 (**A**), Caco-2 (**B**), HT-29 (**C**) and Sk-OV-3 (**D**) cells were subjected to treatment with several concentrations of 7-HC over 24 and 48 h. Data are presented as mean ± SEM from 3 experiments. *Denotes P < 0.05 *vs*. DMSO group.

The cytotoxic effect of 7-HC was examined on several human cancer cell lines. Results showed (Fig. 2) a dose-dependent decrease in cell survival in SF-268, HT-29 and Caco-2 cell lines with almost complete inhibition of proliferation at 25 µg/mL. The IC_50_ values of 7-HC and Cisplatin in all four cancer cell lines are presented in Table 2. Comparable IC_50_ values of 7-HC and Cisplatin were observed for SF-268 and Caco-2 cells. However, while HT-29 displayed resistance to Cisplatin, 7-HC was 8–10 folds more potent with an IC_50_ of 9.1–10.1 µg/mL. Sk-OV-3 cells were the most resistant to treatment with an IC_50_ value >50 µg/mL.

**Table 2:** IC_50_ (µg/mL) values of 7-HC and Cisplatin against different human cancer cell lines.

	Duration				Cell Lines			
	SF-268		Caco-2		HT-29		Sk-OV-3	
	7-HC	Cis	7-HC	Cis	7-HC	Cis	7-HC	Cis
24 h	9.2 ± 0.21	13.9 ± 0.76	9.6 ± 1.40	16.7 ± 2.29	9.1 ± 0.38	>100	>50	>100
48 h	8.1 ± 0.29	5.9 ± 0.32	9.9 ± 0.45	9.3 ± 0.21	10.1 ± 0.36	78.8 ± 5.68	>50	>100

### Synergistic effect of Cisplatin and 7-HC co-treatment

The effect of the co-treatment of 7-HC and Cisplatin on the four cell lines for 24 and 48 h was examined (Table 3). Results suggest that co-treatment of Cisplatin and 7-HC at the highest concentrations (5 and 10 μg/mL respectively) exerted a significant synergistic cytotoxic effect on SF-268, Caco-2 and HT-29 cells as compared to a single treatment of each drug (Tables 4, 5). SF-268 cells were found to be the most sensitive to this combined treatment. The synergistic inhibitory effect of both drugs was not observed on Sk-OV-3 cells.

**Table 3:** Effect of 7-HC, Cisplatin and their combination on human cancer cell survival.

	Treatment (μg/ml)				Cell Lines (% survival)			
	SF-268		Caco-2		HT-29		Sk-OV-3	
	24 h	48 h	24 h	48 h	24 h	48 h	24 h	48 h
Cis (2.5)	90.1 ± 4.6	73.0 ± 1.5	89.8 ± 4.4	87.9 ± 3.1	92.6 ± 3.6	89.7 ± 2.8	97.9 ± 1.9	93.7 ± 1.7
Cis (5)	79.0 ± 4.8	61.7 ± 3.3	68.3 ± 1.9	50.1 ± 2.9	88.9 ± 3.4	88.7 ± 2.3	95.6 ± 2.2	91.7 ± 1.8
7-HC (5)	86.2 ± 2.9	80.1 ± 2.1	85.1 ± 4.1	87.3 ± 3.2	86.8 ± 3.7	89.6 ± 2.7	97.2 ± 2.8	99.1 ± 2.5
7-HC (10)	43.7 ± 2.5	36.8 ± 2.4	49.7 ± 3.8	49.2 ± 3.4	46.5 ± 4.1	54.3 ± 3.0	97.4 ± 3.2	97.3 ± 2.3
Cis (2.5) + 7-HC (5)	81.4 ± 2.4	57.3 ± 1.8^a,b^	78.4 ± 2.1	74.6 ± 3.9	79.9 ± 2.5	82.2 ± 3.9	95.6 ± 1.5	89.4 ± 3.2
Cis (2.5) + 7-HC (10)	35.3 ± 2.3^a^	12.4 ± 3.9^a,b^	41.2 ± 2.5^a^	36.4 ± 2.8^a^	35.6 ± 3.9^a^	44.1 ± 2.8^a,b^	93.9 ± 2.5	88.4 ± 4.1
Cis (5) + 7-HC (5)	66.3 ± 3.2^a,b^	44.1 ± 2.9^a,b^	59.3 ± 4.3^b^	50.9 ± 3.1^b^	76.2 ± 2.8^a^	78.9 ± 3.8	94.0 ± 1.8	86.7 ± 2.6
Cis (5) + 7-HC (10)	26.7 ± 1.9^a,b^	7.7 ± 1.3^a,b^	27.1 ± 2.6^a,b^	23.4 ± 3.9^a,b^	36.6 ± 2.2^a^	40.1 ± 3.5^a,b^	92.8 ± 2.7	87.7 ± 2.8

The values are expressed as mean ± SEM from three independent experiments. Means with superscripts ‘^a^’ and/or ‘^b^’ are significantly (p < 0.05) different compared to similar doses of Cisplatin and/or 7-HC respectively.

**Table 4:** Combination index of 7-HC and cisplatin treatment.

Combination Index (CI)				
Treatment (μg/ml)	SF-268 48 h	Caco-2 48 h	HT-29 48 h	Sk-OV-3 48 h
Combo 1: Cis (2.5) + 7-HC (5)	1.63	1.79	1.87	0.32
Combo 2: Cis (2.5) + 7-HC (10)	0.81	1.18	1.19	0.31
Combo 3: Cis (5) + 7-HC (5)	1.59	1.23	1.87	0.39
Combo 4: Cis (5) + 7-HC (10)	0.68	0.96	1.12	0.52

**Table 5:** Extrapolated CI values at various inhibitory effects.

CI Values at Various Fa (Fa represents % Inhibition/100)																				
*Fa*	0.05	0.1	0.15	0.2	0.25	0.3	0.35	0.4	0.45	0.5	0.55	0.6	0.65	0.7	0.75	0.8	0.85	0.9	0.95	0.97
SF-268	4.5	3.3	2.8	2.4	2.2	2.0	1.8	1.7	1.6	1.5	1.4	1.3	1.2	1.1	1.0	0.9	0.8	0.7	0.6	0.5
Caco-2	3.1	2.5	2.2	2.0	1.8	1.7	1.6	1.5	1.4	1.3	1.2	1.2	1.1	1.0	0.9	0.9	0.8	0.7	0.6	0.5
HT-29	3.7	2.5	2.0	1.8	1.6	1.5	1.4	1.3	1.3	1.2	1.2	1.1	1.1	1.0	0.9	0.9	0.9	0.8	0.7	0.6
SK-OV-3	0.04	0.3	0.9	2.5	5.6	11	21	40	73	99+	99+	99+	99+	99+	99+	99+	99+	99+	99+	99+

### Inhibition of chronic inflammation in rats

**Figure 3:**
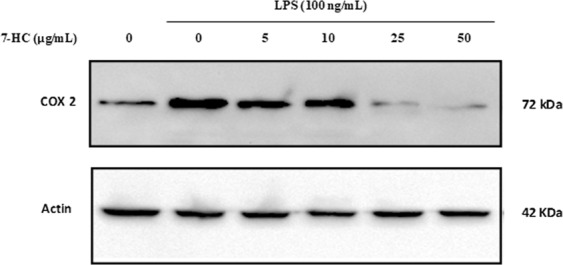
7-HC blocked LPS-induced COX-2 protein expression in rat monocytes (cropped Western Blots). PMBC were incubated with vehicle control alone, with LPS (100 ng/ml) alone, or with 5, 10, 25 or 50 μg/mL of 7-HC for 30 min prior to stimulation with LPS (100 ng/mL) for 6 h (*n* = 3).

7-HC significantly inhibited the chronic inflammation in rats at all concentrations used in a non-dose dependent manner (Table 6). A 100 mg/kg dose of 7-HC showed comparable effects to Diclophenac (10 mg/kg) with 63.1% paw edema inhibition. The effect of increasing concentrations of 7-HC on the level of protein expression of COX-2 in LPS-activated rat peripheral blood mononuclear cells (PBMC) was investigated. Western blot analysis (Fig. 3; cropped Western Blots) demonstrated that 7-HC markedly inhibits the expression of COX-2 compared with the control group (for the full Western Blots please refer to Supplementary Figs S8, S9). Significant suppression of COX-2 protein induction was observed at concentrations of 25 and 50 μg/mL.

**Table 6:** Effect of 7-HC intraperitoneal treatment on formalin-induced chronic inflammation in rats.

Group	Change in paw thickness	% Inhibition
Control (DMSO)	1.25 ± 0.112	0%
Diclophenac (10 mg/kg)	0.498 ± 0.067**	60.2%
7-HC (10 mg/kg)	0.740 ± 0.051**	40.9%
7-HC (25 mg/kg)	0.760 ± 0.123**	39.3%
7-HC (50 mg/kg)	0.304 ± 0.075*	75.7%
7-HC (100 mg/kg)	0.462 ± 0.121**	63.1%

*Significant difference (*p* < 0.05) with respect to the control.

### Activity of 7-HC against rat monocytes

**Figure 4:**
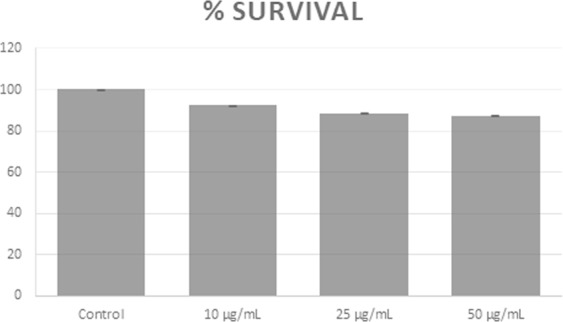
Activity of 7-HC using WST against isolated rat monocytes at 6 h post-treatment.

The cytotoxic effect of 7-HC using WST (Fig. 4) was tested on isolated monocytes after 6 h of treatment. The results revealed that 2-himachalene-7-ol causes only 7.7%, 11.5% and 12.7% reduction in cell survival at concentrations of 10 µg/mL, 25 µg/mL and 50 µg/mL, respectively.

## Discussion

Phytochemical analysis of the CLOE revealed that sesquiterpenes are the main constituents with 7-HC (40%), α-himachalene (2.96%), β-himachalene (3.18%) and γ-himachalene (3.37%) being the major components. A comparable qualitative composition of the aforementioned sesquiterpenes was previously reported in the Lebanese cedar wood oil^21^. However, in the present study, the percentage composition of α-himachalene, β-himachalene and γ-himachalene present in CLOE was lower than that of heartwood oil previously reported^21^. This quantitative difference may be attributed to factors such as geographical location, humidity, altitude level, collection period, extraction technique and plant age.

In the present study, we have optimized a chromatographic procedure which enables the isolation of 7-HC from CLOE in excess of 97% purity. The purified sesquiterpene was then evaluated for its *in vitro* cytotoxic effects against several types of cancer cells as well as its *in vivo* and *in vitro* anti-inflammatory activity. The results showed that 7-HC exerts a dose dependent cytotoxicity against all tested cancer cells except for Sk-OV-3, indicating selectivity against certain cancer cell lines. This anticancer effect suggests that the previously reported activity of CLOE against human leukemia cells could be attributed to 7-HC^11^. In addition, earlier work in our lab showed that β-2-himachalene-6-ol, an isomer of 7-HC, isolated from wild carrot demonstrated potent anticancer activity against several human and murine cancer cell lines^22,23^. β-2-Himachalene-6-ol was also found to exhibit significant anti-tumor promoting effect against DMBA/TPA skin carcinogenesis and dimethylhydrazine-induced colon carcinogenesis in mice^24^.

Cisplatin, the most commonly used antineoplastic agent, mediates its anticancer activity through various cytotoxic mechanisms, including DNA damage, activation of apoptotic pathways and inflicting damage to the cells through inflammation and oxidative stress^25^. The current results showed that HT-29 cells were resistant to Cisplatin (IC_50_ > 100 µg/mL and 78.78 µg/mL at 24 and 48 h, respectively), while 7-HC exhibited potent dose-dependent anticancer activity (IC_50_ = 9.122 µg/mL and 10.11 µg/mL at 24 and 48 h, respectively). Sk-OV-3 cells, however, were shown to be resistant against both 7-HC and Cisplatin treatment (IC_50_ >100 µg/mL). The IC_50_ values of Cisplatin on the same cancer cell lines are in agreement with the literature^26,27^. In an attempt to achieve an improved therapeutic outcome, all 4 cell lines were subjected to a combination of 7-HC and the conventional antineoplastic drug Cisplatin (Table 3). Drug Combination Index (CI) was determined using the commonly used Loewe additivity model and CompuSyn software^28^. Briefly, CI values equal to 1 suggest additivity, values below 1 suggest synergy while values above 1 suggest antagonism^28^. Table 4 shows that the most desired combinations which lead to synergism correspond to Combination 2: Cis(2.5) + 7-HC(10) and Combination 4: Cis(5) + 7-HC(10). In addition, the Loewe model extrapolates^28^ (Table 5) that synergism would occur with combinations of 7-HC and Cisplatin that lead to 75% cell inhibition (SF-268, Caco-2 and HT-29) or above. Results reveal that combinations consisting of a high 7-HC concentration (10 µg/mL) and a low Cisplatin concentration (2.5 µg/mL) would induce significant cell inhibition via synergistic pathways. Such a synergistic effect could be exploited in reducing the adverse effects of Cisplatin by eliminating the dose limiting nephrotoxicity while maintaining potency against cancerous cells^25^. Table 4 also shows that all four drug combinations exerted a synergistic effect against the Sk-OV-3 cell line, yet these results are insignificant as cell inhibition via synergism didn’t exceed 15%. As the literature reports, synergistic effects are mainly desirable when accompanied with significant cell inhibition as observed with Combinations (2) and (4) against SF-268, Caco-2 and HT-29 cell lines^28^.

A substantial body of evidence obtained from both *in vivo* and *in vitro* studies reported that plant-derived extracts containing sesquiterpenes, alkaloids, phenolic compounds and flavonoids demonstrate anti-inflammatory activity by controlling the levels of various inflammatory biomarkers including TNF-α, NF-κB, NO, iNOS and COX-2^29^. Cyclooxygenase-2 (COX-2) is a key pro-inflammatory enzyme induced by LPS or cytokines, and was found to be implicated in inflammation, malignancies, and angiogenesis^30^. Therefore, identifying a new compound with an inhibitory effect on COX-2 protein is regarded as an important requirement for the treatment of inflammation-related disorders. The results of this study show that 7-HC possesses significant anti-inflammatory activity against chronic inflammation in rats. Several inflammatory mediators such as kinins, prostaglandins, and serotonin may account for the edema formation caused by sub plantar formalin injection^31^. The increased synthesis of prostaglandins could be due to increased release of arachidonic acid from the membrane phospholipids and/or the up-regulation of COX-2. Western blot analysis showed that the production of COX-2 was markedly elevated after LPS treatment of rat monocytes. However, the application of different doses of 7-HC inhibited the protein expression of COX-2 by LPS in a dose dependent manner. Hence, the anti-inflammatory activity of 7-HC is partially mediated through the suppression of COX-2 expression. Previous reports revealed that himachalol, derived from the wood of *C. deodara* displayed substantial anti-allergic activity and has been identified as a major anti-spasmodic constituent^32^. The cytotoxic effect of 7-HC against rat monocytes was shown to be insignificant with 7.7%, 11.5% and 12.7% reduction in cell survival at concentrations of 10 µg/mL, 25 µg/mL and 50 µg/mL, respectively. This indicates that 7-HC has a minor cytotoxic effect on isolated rat monocytes (at the selected concentrations). This result also supports the selectivity of 2-himachalene-7-ol on cancer cells relative to normal cells.

## Conclusion

*C. libani* essential oil was extracted and analyzed via GCMS whereby 2-himahcalene-7-ol (7-HC) was found to constitute 40% of the oil. 7-HC was isolated using silica-gel column chromatography to afford the sesquiterpenes as a white crystalline solid and 97% purity. 7-HC demonstrated potent cytotoxic activity against SF-268 (IC_50_ 8.1 μg/mL), HT-29 (IC_50_ 10.1 μg/mL) and Caco-2 (IC_50_ 9.9 μg/mL). In addition, while HT-29 displayed resistance to Cisplatin, 7-HC was 8–10 folds more potent. Co-treatment with combinations of 7-HC:Cisplatin (2:1 and 4:1 ratio) showed a significant synergistic anti-proliferative effect against SF-268, HT-29 and Caco-2 cells. 7-HC also exhibited significant anti-inflammatory effect in formalin-induced paw edema in rats as well as dose dependent inhibition of LPS-induced COX-2 protein expression in isolated rat monocytes. The present study demonstrates that 7-HC possesses promising anticancer and anti-inflammatory activities, and may serve as a lead molecule in cancer therapy.

## Materials and Methods

### General experimental procedures

UV spectra were recorded using a Shimadzu UV2450 spectrophotometer. The ^1^H, ^13^C and 2D NMR spectra were obtained on a Bruker Avanc3–400 MHz NMR spectrometer or Varian Mercury-300 NMR spectrometer using TMS as an internal reference. TLC analysis was carried out on silica gel plates (ACROS organics, New Jersey, USA). Silica gel 60 (230–400 mesh or 70–230 mesh, 47 cm by 2.5 cm, ACROS organics, New Jersey, USA) was used for column chromatography. GCMS analysis was carried out using Hewlett Packard, HP6890 series, fitted with a fused silica HP5-MS 5% phenyl methyl siloxane cap column (30 m × 0.25 mm i.d., film thickness 0.25) and directly coupled to the MS. The ELIZA microplate reader was purchased from BioTek (Winooski, VT, USA).

### Chemicals and reagents

Dulbecco’s modified Eagle’s medium (DMEM) and dimethyl sulfoxide (DMSO) were purchased from Sigma (St. Louis, Missouri, USA). PVDF membranes were purchased from Pall Corporation (Ann Arbor, MI, USA). Actin and Cox-2 antibodies were purchased from Abcam (Cambridge, MA, USA). HRP-coupled secondary antibodies were purchased from Promega Corporation (Madison, WI, USA). The ECL kit was purchased from Abcam (Milton, Cambridge, UK). Fetal bovine serum (FBS), penicillin-streptomycin, glycine, lysis buffer solution, phosphate buffer saline (PBS), bovine serum albumin (BSA), tris-buffered saline with Tween 20^®^ (TBST), HEPES buffer, sodium dodecyl sulfate (SDS), well plates, pentane, diethyl ether, hexane, ethyl acetate, anisaldehyde, ethanol, paraformaldehyde, trypan blue, Ficoll (Histopaque-1077). Crystal violet, trypsin, d_6_-DMSO and CDCl_3_ were purchased from Sigma (St. Louis, Missouri, USA) unless otherwise stated.

### *Cedrus libani* extraction

Cedar wood was collected from North Lebanon, from mature cedar trees during the month of February when the trees are pruned in order to enhance their growth in the following season. Pieces of pruned wood were collected after acquiring permission from the Municipality of Amioun, El-Koura. The tree was identified according to the characteristics described in the “Medicinal Plants of The World”^33^. A specimen, (ID 2015-0019) was deposited at the School of Arts and Sciences, Lebanese American University, Lebanon. The wood sample was air dried in the shade, shredded and subjected to hexane extraction (72 h). The filtered extract was evaporated to dryness under reduced pressure and the remaining oil (CLOE) was dried over anhydrous sodium sulfate (yield 1.95%) and stored in a closed amber bottle at 4 °C until use. CLOE (5 g) was chromatographed on a silica gel column (230–400 mesh) using a gradient mobile phase of hexane/ethyl acetate to collect three fractions (9:1, 600 mL, F1; 8:2, 600 mL, F2; 7:3, 500+ mL, F3). Fractions were analyzed by TLC using hexane:ethyl acetate (8:2) as mobile phase and plates were stained with 2% anisaldehyde in which 2-himachelen-7-ol appeared as a pink spot with an *Rf* value of 0.78. F2 (1.8 g) was then mixed with acetonitrile and stored for 3 days in the freezer to recrystallize 2-himachelen-7-ol as a white crystalline solid (1.2 g, 97% purity). ^13^C NMR 75 MHz in CDCl_3_: δ 133.4, 125.6, 76.2, 51.9, 43.6, 41.4, 38.4, 36.4, 33.5, 32.7, 31.7, 26.7, 23.6, 22.3 and 19.9 ppm.

### Gas Chromatography and Mass Spectrometry (GC-MS) analysis

CLOE, fractions and 7-HC were analyzed via GC-MS using helium as the carrier gas with splitless injection and a flow rate of 1.2 mL/min. The temperature program was 2.0 min at 70 °C, from 70 to 130 °C at 8 °C/min and hold for 5 min, from 130 to 180 °C at 2 °C/min and hold for 10 min, from 180 to 220 °C at 15 °C/min and hold for 2 min and then from 220 to 280 °C at 15 °C/min and hold for 22 min. Preliminary identification of all compounds was performed by comparing their mass spectra with the literature (NIST11 and Wiley9). Percentage composition was computed from GC peak areas. GC-MS: CHCl_3_ *m/z*: 222.2 (1), 204.2 (46), 189.2 (17), 175.1 (4), 161.1 (20), 147.1 (16), 133.1 (34), 119.1 (100), 105.1 (44), 91.1 (41), 77.1 (21), 69.1 (15) and 55.1 (16).

### Cell survival assay

Four human cancer cell lines were used in this study: SF-268 (human astrocytoma cell line), Caco-2 (human colon cancer cell line), HT-29 (human colon cancer cell line), and Sk-OV-3 (human ovarian cancer cell line). SF-268, Sk-OV-3 were grown in Dulbecco’s modified Eagle’s medium (DMEM), while Caco-2 and HT-29 were maintained in Roswell Park Memorial Institute Medium (RPMI). Both media contained 10% Fetal Bovine Serum (FBS) and 100 μg/mL streptomycin and 100 U/mL penicillin. All cell lines were incubated in a humidified chamber at 37 °C and 5% CO_2_. Cells (1 × 10^4^ cell/mL) were plated in 96-well plates for 24 h and then treated with either 7-HC in DMSO (1, 5, 10, 15 and 25 μg/mL) or cisplatin for 24 h and 48 h. In addition, the concomitant treatment of 7-HC (2.5 and 5 μg/mL) and Cisplatin (5 and 10 μg/mL) was carried out. Cell viability was measured using WST-1 (Roche Diagnostics, Indianapolis, IN, USA). Absorbance was measured at 450 nm using Multiskan FC microplate ELISA reader (Thermo fisher Scientific, Rockford, IL, USA). Cells were plated in duplicates, and experiments were repeated three times.

### Monocytes isolation and stimulation

The experiment was conducted in accordance with the internationally accepted principles set by the Office of Laboratory Animal Welfare (NIH, PHS Policy on Human Care and Use of Laboratory Animals, USA 2015) and approved by the Animal Ethical Committee at the Lebanese American University. Isolation of fresh peripheral blood mononuclear cells (PBMCs), consisting of monocytes and lymphocytes, was performed as previously described^26^ from blood samples of healthy young male Sprague Dawley rats of (6 weeks age; weighing 250 g) and pretreated with EDTA. EDTA-treated blood (4 mM) was diluted with an equal sterile volume of warm PBS, and centrifuged over Ficoll. The buffy coat that formed at the interface and containing most of the monocytes cells is carefully removed with a sterile pipet and transferred into a sterile 50 ml conical tube and washed twice with sterile warm PBS by centrifugation. Cells were then seeded in 2 mL RPMI medium supplemented with FBS (10%), penicillin (100 U/mL) and streptomycin (100 μg/mL) in six-well culture plates (4 × 10^5^ cells/cm^2^) and allowed to adhere overnight (37 °C; 5% CO_2_). The non-adherent cells (mainly lymphocytes) were removed by vigorous washing with warm sterile PBS (three times). The obtained monocytes/macrophages exceeded 95% purity. Trypan blue exclusion was used to determine cell viability. Trypan blue exclusion was used to determine cell viability. Monocytes were cultured in RPMI-free media in a total volume of 2 mL for 6 h in six-well plates in the presence or absence of bacterial LPS (100 ng/mL) and increasing concentrations of 7-HC (5, 10, 25, 50 μg/mL). 7-HC was added 30 min before stimulation with LPS.

### Western blot analysis

PBMCs were collected on ice, washed with PBS, lysed with lysis buffer and centrifuged at 12,000 g for 10 min at 4 °C. The cell lysate was heated at 100 °C for 5 min, and the protein content was determined using the Bio-Rad protein assay (Bio-Rad, Hercules, CA, USA). Equal concentrations of the proteins were loaded to 10% SDS-PAGE and then transferred to PVDF membrane (Pall Corporation, Ann Arbor, USA) and blocked with blocking buffer (1 × TBS, 0.1% Tween-20, 5% skim milk) for 2 h. The membranes were then probed with primary antibodies against Actin and Cox-2 at 4 °C overnight. The primary antibodies were then washed away with TBST for 2 h and the membranes were treated with horseradish peroxidase (HRP)-coupled secondary antibodies for 1 h, and washed with TBST afterwards. Protein detection was performed using the chemiluminescence ECL kit. Finally, blot images were then obtained with the image lab Software (BioRad, Chemidoc imaging instrument).

### Monocytes cell survival assay

Rat monocytes were plated in 12-well plates in RPMI medium supplemented with FBS (10%), penicillin (100 U/mL) and streptomycin (100 μg/mL) and allowed to adhere overnight (37 °C; 5% CO_2_) for 24 h. Cells were then treated with increasing concentrations of 7-HC (10, 25 and 50 μg/mL) for 6 h. Cell viability was measured using WST-1 (Roche Diagnostics, Indianapolis, IN, USA). Absorbance was measured at 450 nm using Multiskan FC microplate ELISA reader (Thermo fisher Scientific, Rockford, IL, USA). Cells were plated in duplicates, and experiments were repeated three times.

### Formalin induced paw edema

Sprague Dawley rats were divided into six groups of six animals each. In all groups, chronic inflammation was produced by a subplantar injection of 20 µL of 2% formalin in the right hind paw^34^. Thirty min prior to formalin injection, four groups received 7-HC (i.p.) in saline at a concentration of 10, 25, 50, or 100 mg/kg BW, one group received the standard reference drug diclofenac (10 mg/Kg BW, i.p.), and one group served as a negative control. The administration of 7-HC and diclofenac was continued once daily for 6 consecutive days. The paw thickness was measured using a Vernier caliper before and 6 days after formalin injection^31^. The increase in paw thickness was calculated using the formula: [P_t_ – P_0_]; where P_t_ is the thickness of paw at 6 days after formalin injection and P_0_ is the paw thickness at time 0. The percent inhibition was calculated using the formula: [(C − T)/C × 100]; where *C* is the increase in paw thickness of the positive control and *T* is that of treatments.

### Statistical analysis

The results were analyzed for statistical significance using one way analysis of variance (ANOVA). Values of the different tested parameters within each group are presented as mean ± SEM. All data were analyzed with the statistical package SPSS 18, and differences between groups were considered statistically significant if p-value < 0.05. The IC_50_ values were calculated using the nonlinear regression curve with the use of Graph Pad Prism version 5.0 software for Windows.

## Supplementary information

In Vitro and In Vivo Evaluation of the Anticancer and Anti-inflammatory Activities of 2-Himachelen-7-ol isolated from Cedrus Libani

## Data Availability

The authors declare that the manuscript contains the minimal dataset that is required to interpret, replicate and build upon the methods and findings reported in the article.
