# Supplementary material for: In Vitro and In Vivo Evaluation of the Anticancer and Anti-inflammatory Activities of 2-Himachelen-7-ol isolated from Cedrus Libani
Source: Sci Rep. 2019 Sep 6;9:12855. doi: 10.1038/s41598-019-49374-9 (PMC6731217; doi:10.1038/s41598-019-49374-9)
Supplement: Supplementary file 1 — In Vitro and In Vivo Evaluation of the Anticancer and Anti-inflammatory Activities of 2-Himachelen-7-ol isolated from Cedrus Libani [file 41598_2019_49374_MOESM1_ESM.docx]

**Supplementary**

***In Vitro* and *In Vivo* Evaluation of the Anticancer and Anti-inflammatory Activities of 2-Himachelen-7-ol isolated from *Cedrus Libani***

Andree Elias,^a^ Wassim N. Shebaby,^a^ Bilal Nehme,^a^ Wissam Faour,^b^ Bassem S. Bassil,^c^ Joelle El Hakim,^a^ Rita Iskandar,^a^ Nahia Dib-Jalbout,^a^ Mohamad Mroueh,^d^ Costantine Daher,^a^ and Robin I. Taleb^a*^

*^a^Department of Natural Sciences, Lebanese American University, Byblos1102 2801, Lebanon*

*^b^School of Medicine, Lebanese American University, Byblos1102 2801, Lebanon*

*^c^Faculty of Arts and Sciences, University of Balamand, PO Box 100, Tripoli, Lebanon*

*^d^School of Pharmacy, Lebanese American University, Byblos 1102 2801, Lebanon*

^*^Corresponding author:

Robin Taleb, PhD

Lebanese American University

School of Arts and Sciences, Department of Natural Sciences

Byblos, Lebanon, P.O Box 36

Tel: +961 9 547 262; Ext: 2429

Fax: +9619546262

robin.taleb@lau.edu.lb

Supplementary Information | **Figures**

Supplementary Figure S1 **|** GC Chromatogram of CLOE.

Supplementary Figure S2 **|** GC Chromatogram of F2.

Supplementary Figure S3 | GC Chromatogram of 2-Himachalen-7-ol.

Supplementary Figure S4 | MS of 2-Himachalene-7-ol.


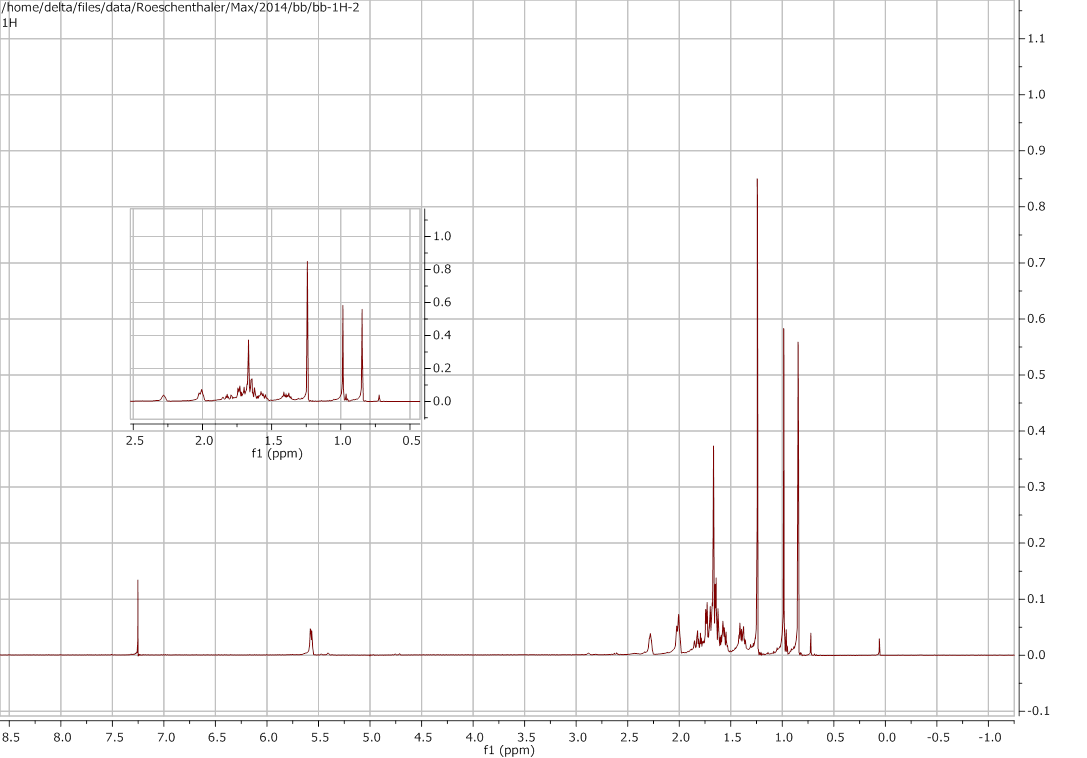


Supplementary Figure S5 | ^1^H NMR (400 MHz, CDCl_3_) spectrum of 2-Himachalene-7-ol.

Supplementary Figure S6 | ^13^C NMR (400 MHz, CDCl_3_) spectrum of 2-Himachalene-7-ol.

Supplementary Figure S7 | DEPT (400 MHz, CDCl_3_) spectrum of 2-Himachalene-7-ol.


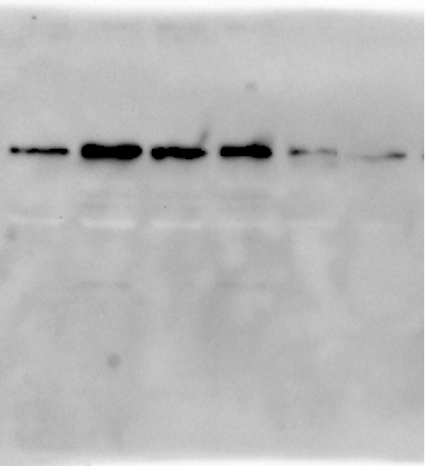


Supplementary Figure S8 | 7-HC blocked LPS-induced COX-2 protein expression in rat monocytes (Full Western Blot). PMBC were incubated with vehicle control alone, with LPS (100 ng/ml) alone, or with 5, 10, 25 or 50 μg/mL of 7-HC for 30 min prior to stimulation with LPS (100 ng/mL) for 6 h (*n* = 3).


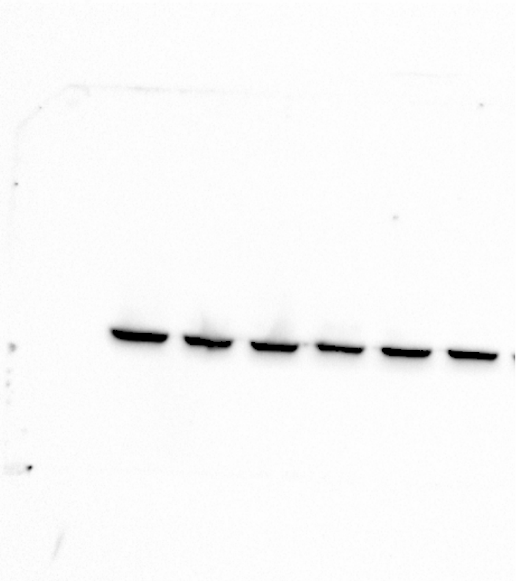


Supplementary Figure S9 | 7-HC blocked LPS-induced Actin protein expression in rat monocytes (Full Western Blot). PMBC were incubated with vehicle control alone, with LPS (100 ng/ml) alone, or with 5, 10, 25 or 50 μg/mL of 7-HC for 30 min prior to stimulation with LPS (100 ng/mL) for 6 h (*n* = 3).
